# Successful customer intercept interview recruitment outside small and midsize urban food retailers

**DOI:** 10.1186/s12889-016-3717-2

**Published:** 2016-10-05

**Authors:** Jennifer E. Pelletier, Caitlin E. Caspi, Liana R. N. Schreiber, Darin J. Erickson, Lisa Harnack, Melissa N. Laska

**Affiliations:** 1 Division of Epidemiology and Community Health, University of Minnesota, 1300 S. Second St., Suite 300, Minneapolis, MN 55454 USA; 2 Department of Family Medicine and Community Health, University of Minnesota, 717 Delaware St SE, Minneapolis, MN 55414 USA

**Keywords:** Research design in epidemiology, Measurement, Nutrition, Health promotion, Diet

## Abstract

**Background:**

Customer intercept interviews are increasingly used to characterize food purchases at retail food outlets and restaurants; however, methodological procedures, logistical issues and response rates using intercept methods are not well described in the food environment literature. The aims of this manuscript were to 1) describe the development and implementation of a customer intercept interview protocol in a large, NIH-funded study assessing food purchases in small and midsize food retailers in Minneapolis and St. Paul, Minnesota, 2) describe intercept interview response rates by store type and environmental factors (e.g., neighborhood socioeconomic status, day/time, weather), and 3) compare demographic characteristics (e.g., gender, race/ethnicity) of participants versus non-participants.

**Methods:**

After a pilot phase involving 28 stores, a total of 616 interviews were collected from customers exiting 128 stores in fall 2014. The number of eligible customers encountered per hour (a measure of store traffic), participants successfully recruited per hour, and response rates were calculated overall and by store type, neighborhood socio-economic status, day and time of data collection, and weather. Response rates by store type, neighborhood socio-economic status, time and day of data collection, and weather, and characteristics of participants and non-participants were compared using chi-square tests.

**Results:**

The overall response rate was 35 %, with significantly higher response rates at corner/small grocery stores (47 %) and dollar stores (46 %) compared to food-gas marts (32 %) and pharmacies (26 %), and for data collection between 4:00–6:00 pm on weekdays (40 %) compared to weekends (32 %). The distribution of race/ethnicity, but not gender, differed between participants and non-participants (*p* < 0.01), with greater participation rates among those identified as Black versus White.

**Conclusions:**

Customer intercept interviews can be successfully used to recruit diverse samples of customers at small and midsize food retailers. Future community-based studies using customer intercept interviews should collect data sufficient to report response rates and consider potential differences between the racial/ethnic composition of the recruited sample and the target population.

## Background

Customer intercept (CI) interviews are an increasingly common data collection method for the study of obesity-related policies and programs. They have been used to characterize retail food/beverage purchases at small food stores [1–4] and measure the impact of healthy corner store interventions [5, 6], menu calorie labeling in fast food restaurants [7–10], and food labeling and taxation experiments in a hospital cafeteria [11]. Yet the methodological procedures, feasibility, and conditions for successful recruitment of participants for CI interviews in community settings are not well described in the literature.

To our knowledge, only three published studies in the food environment literature have reported response rates from CI interviews of food/beverage purchases, and all were conducted with adult shoppers in New York City. Reported response rates were 55.2 % outside fast food chain restaurants [10], 32.9 % outside corner stores across all areas of the city [12], and 53 % at baseline and 63 % at follow-up outside corner stores in high poverty neighborhoods [3]. None of these studies collected information on non-participants, such as gender or race/ethnicity, to evaluate potential non-response bias in the recruited sample. If customers who participate in CI interviews differ systematically from customers who refuse (e.g., if they are more likely to purchase healthy foods/beverages or disproportionately represent some demographic groups), the resulting estimates of purchasing patterns could be biased. In addition, no prior studies examined how recruitment rates varied by factors such as time of day or neighborhood socioeconomic status.

Given the scant literature on CI interview methods for assessing food/beverage purchases, the aims of this paper were to 1) describe methodology development and implementation in a study assessing food and beverage purchases in small and midsize food retailers in Minneapolis and St. Paul, Minnesota; 2) assess how response rates differed by type of store, neighborhood socioeconomic status (SES), time of day, and weather; and 3) examine demographic characteristics of participants and non-participants. Results may be used to inform protocol development of future community-based studies.

## Methods

The STORE (STaple Foods ORdinance Evaluation) Study is a large-scale evaluation of a local Staple Foods Ordinance, passed by the Minneapolis City Council in 2008 and revised in 2014 (1R01DK104348, 3U48DP005022; PI: M. Laska). The revised ordinance requires licensed grocery stores to stock minimum quantities and varieties of products in 10 categories of staple foods and beverages beginning April 2015. CI protocol development to assess purchasing in affected food retailers occurred in the summer of 2014. Baseline data collection occurred during an 8-week period from September to November 2014 outside small and midsize food retailers, including corner/small grocery stores, food-gas marts, dollar stores, and pharmacies in Minneapolis and nearby St. Paul (a comparison site). Data were collected on types and cost of foods/beverages purchased, customers’ frequency and reasons for shopping at the store, their perceptions of neighborhood healthy food access, and demographics.

### Baseline store selection

A list of licensed grocery stores was obtained from the relevant licensing agencies for Minneapolis (Minneapolis Health Department) and St. Paul (Minnesota Department of Agriculture). Minneapolis stores and comparable St. Paul stores were eligible for the evaluation if the Staple Foods Ordinance would have applied to them. Ineligible stores included those that sold food/beverages as an accessory use to their primary business and did not participate in the Supplemental Nutrition Assistance Program (e.g., bakeries/delis, spice shops) and stores that were located in the core downtown commercial districts (which were not expected to stock a wide array of staple foods). Supermarkets and Special Supplemental Nutrition Program for Women, Infants and Children (WIC)-participating stores were also ineligible because they were presumed to already meet the revised ordinance requirements [13, 14]. Fifteen stores with invalid licensing addresses were also excluded. Of 255 eligible stores, 90 stores per city were randomly selected for this sample. Twenty of these stores were determined to be ineligible upon visiting the store for data collection (5 participated in WIC, 1 supermarket, 7 accessory use, 2 did not sell food, and 5 out of business). Of the remaining 160 stores, 128 (80 %) allowed study staff to conduct CI interviews outside the store.

### Interview protocol

In summer 2014, the study team adapted existing protocols to suit the needs of STORE study design and community context [2, 8]. The adapted protocol was approved by the University of Minnesota Institutional Review Board and piloted over 4 weeks in 28 stores. Areas for protocol improvement were discussed at weekly team meetings. There were no changes to the interview following the pilot, but minor protocol changes, such as collecting data at various times throughout the day and recording various amounts of information on non-participants, were identified by the team and subsequently piloted.

In pairs, data collectors entered a store, introduced themselves as part of a university public health research team, and explained they would be asking shoppers exiting the store to participate in a brief interview. They emphasized they would not disrupt the flow of business or block the store entrance/exit. If the owner/manager/employee agreed to data collection, data collectors asked if the cashier (s) would be willing to tell customers they were recruiting interview participants outside. Data collectors maintained discretion to cancel data collection shifts due to inclement weather (e.g., rain, cold temperatures) and to leave a store if safety concerns arose. Additional protocol details appear in Table 1.

**Table 1:** Key components of STORE study customer intercept interview protocol

Staffing
• Teams of two data collectors
• 2–4 hour data collection shifts
• Shifts scheduled between 9:00 am and 8:00 pm, 7 days per week
• Active data collection at each store lasted 30–45 min
Recruitment
• Data collectors asked permission from store owners, managers, or employees to conduct data collection
• Data collectors stood outside the store on either side of the primary exit or between the exit and the parking area
• Data collectors wore t-shirts with the university’s logo and colors and wore a university identification badge on a lanyard around their neck
• Data collectors held clipboards to their chest with a full-page, color recruitment flyer attached to the back that customers could see as they passed
• All adults with a visible food, beverage, or bag of purchases were invited to participate (visual eligibility screen)
• If a group left the store together, all adults in the group with visible purchases were approached and all who were interested were invited to participate
• Data collectors followed a recruitment script, conducted a verbal eligibility screen, read an informed consent statement, and gave participants written information about the study
• Reason for ineligibility or refusal, apparent gender, and apparent race/ethnicity were recorded for non-participants
Data Collection
• Data collectors conducted the interview verbally and recorded detailed information on each food and beverage item purchased (product name, size, quantity, price paid)
• If a participant did not have a receipt for their food/beverage purchase, data collectors re-entered the store at the end of the visit to verify prices
Participation Incentive
• Participants were given a $10 gift card after completing the interview

### Participant recruitment

Eligible customers were ≥18 years old, spoke English well enough to respond to interview questions, and had just made a food or beverage purchase. During the pilot, a notable proportion of individuals leaving stores were not purchasing foods/beverages, particularly at food-gas marts, dollar stores, and pharmacies; they were instead purchasing items such as newspapers, gasoline, tobacco, or home goods. A visual eligibility assessment (e.g., presence of a visible food, beverage, or bag of unknown purchases) prior to recruiting each individual was thus added to the protocol.

Data collectors held clipboards with a full-page recruitment flyer on the back and approached all adults carrying a food, beverage and/or bag of purchases by extending a quick invitation (“Hi! Do you have 5 min to get $10?”). If the customer hesitated, data collectors extended additional encouragement (e.g., “it’s really quick”, “it’s fun!”) to start a conversation and describe the study. Customers were invited to participate using a brief recruitment script, verbally screened for eligibility, read an informed consent statement, and given written information about the study. Data collectors administered the interview verbally, asked to look at customers’ food/beverage purchases and receipts (when available) to collect detailed product information on foods/beverages purchased, and gave participants a $10 gift card. Each interaction lasted approximately 5 min.

If data collectors did not recruit any participants at a store after 30 min, they thanked the store employee and left. If they recruited at least 1 participant, they stayed an additional 15–30 min. Data collectors were instructed to leave a store if they suspected people were purchasing items only to receive a gift card for their participation. Data collectors recorded the start and end time of data collection at each store. The target number of recruited participants at each store was at least 5. Stores where 1–4 participants were recruited during a single visit were visited again on a different day to recruit additional participants.

For each individual who exited the store but did not complete a interview, data collectors recorded the reason for non-participation (e.g., ineligible because under 18; ineligible because non-English speaker; ineligible because no food, beverage, or store bag purchase (based on visual and/or verbal assessment); or refused/no response) and the person’s apparent gender (male, female, don’t know) and presumed race/ethnicity (White/Caucasian, Black/African American, Asian, Hispanic, don’t know). Customers who were not approached because they were clearly under age 18 were not recorded on the form. Non-English speakers were documented if a language barrier was identified after interacting with an individual (e.g., if a child had to translate the invitation for their parent). If the individual appeared to be eligible (adult with a visible food, beverage, or store bag purchase) but ignored the data collector or said they were not interested, data collectors marked them as refusals. Data collectors did not record non-participants who exited the store while both data collectors were busy conducting interviews.

### Measures

Each recruitment encounter was classified as eligible or ineligible based on the visual and verbal eligibility screens. To be conservative, individuals who refused to be screened verbally (i.e., those who did not respond to data collectors) but appeared eligible based on visual eligibility screening were considered eligible. Response rates were calculated as the number of completed interviews divided by eligible customers. The number of eligible customers encountered per hour (a measure of store traffic), participants successfully recruited per hour, and response rates were calculated overall and by store type, neighborhood SES, day and time of data collection, and weather.

Each store was classified by data collectors in the field as a corner/small grocery store (*n* = 57), food-gas mart (*n* = 39), dollar store (*n* = 11), pharmacy (*n* = 20), or general retailer (*n* = 1). The general retail store was excluded from analyses stratified by store type but was included in all other analyses. A dichotomous measure of neighborhood SES was developed based on the percent of families living below 185 % of the federal poverty level (e.g., the maximum allowed under federal WIC eligibility guidelines) in the census tract in which each store was located [15]. Census tracts in which ≤50 % of families lived below this poverty threshold were classified as higher SES neighborhoods; census tracts in which >50 % of families lived below this poverty threshold were classified as lower SES neighborhoods.

Each store visit was categorized based on the day and time data collection began: weekday (Monday-Friday) mornings (9:00 am-10:59 am), weekday midday (11:00 am-12:59 pm), weekday mid-afternoon (1:00 pm-3:59 pm), weekday rush hour (4:00 pm-5:59 pm), and weekend (Saturday-Sunday, 10:30 am-6:59 pm). Weather data from Minneapolis-St. Paul International Airport were downloaded from the National Oceanic and Atmospheric Administration [16]. Daily temperature midpoints were calculated based on daily high and low temperatures and categorized as <10 °C, 10–15.5 °C, ≥15.6 °C. Each data collection day was also dichotomized based on the presence of precipitation (≥0.025 cm).

### Analysis

Response rates by store type, neighborhood SES, time and day of data collection, and weather, and characteristics of participants and non-participants were compared using chi-square tests (with two-tailed α = 0.05 used to determine statistical significance). Analyses were completed in 2015 using Stata 13.1 (Stata Corp, College Station, TX).

## Results

**Fig. 1:**
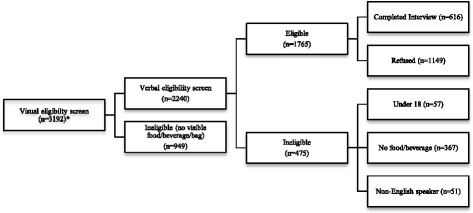
Customer intercept interview recruitment results

Data collectors made a total of 204 visits to 128 stores. Participants recruited during a single store visit ranged from 0 to 15 (median = 3). The mean length of time of data collection for each store visit was 40 min. An estimated 55 % of individuals exiting stores were eligible for the study based on visual and verbal eligibility screens (Fig. 1). Approximately 30 % (*n* = 949) of individuals exiting the stores did not have a visible food, beverage, or store bag purchase. Of those that passed visual eligibility screens, 21 % (*n* = 475) were determined to be ineligible after verbal screening. Over 75 % of customers who exited the store carrying a bag of purchases but were ultimately deemed ineligible for the study were ineligible because they did not make a food/beverage purchase (367/475). The remaining 1765 customers were considered eligible. Of these, 616 participated for an overall response rate of 35 %.

### Response rates

On average, 4.5 interviews per hour were collected and 13 eligible customers per hour were encountered exiting the stores (Table 2). The number of eligible customers that exited food-gas marts and pharmacies was over twice as high as the number at corner/small grocery stores (17 versus 7 per hour); however, these stores also had significantly lower response rates than corner/small grocery stores and dollar stores. The number of interviews collected per hour was highest at food-gas marts (5.6) and lowest at corner/small grocery stores (3.4).

**Table 2:** Response rates by store and shift characteristics

	Number Eligible/ Hour	Surveys Collected/ Hour	Response Rate	
Total	13	4.5	35 %	
Store Type^a^				
Corner/small grocery store	7	3.4	47 %	a
Food-gas mart	17	5.6	32 %	b
Dollar Store	11	5.0	46 %	a
Pharmacy	17	4.5	26 %	c
Neighborhood SES^b^				
Higher	12	4.3	35 %	a
Lower	14	5.0	36 %	a
Data Collection Day and Start Time				
Weekday morning (9:00 am-10:59 am)	11	4.0	36 %	ab
Weekday mid-day (11:00 am-12:59 pm)	14	4.5	33 %	ab
Weekday mid-afternoon (1:00 pm-3:59 pm)	13	4.7	36 %	ab
Weekday rush hour (4:00 pm-5:59 pm)	14	5.5	40 %	a
Weekend (10:30 am - 16:59 pm)	13	4.1	32 %	b
Weather on Day of Data Collection				
Temp Midpoint < 10 °C	11	4.8	34 %	a
Temp Midpoint 10–15.5 °C	12	4.3	36 %	a
Temp Midpoint ≥15.6 °C	13	4.5	35 %	a
No Precipitation	13	4.6	35 %	a
Any Precipitation	12	4.3	36 %	a

Notes: Response rates that share a letter within each categorical variable are not significantly different at *p* < 0.05

^a^Data collected at one general retail store were excluded from the analysis by store type

^b^SES is socioeconomic status. Higher SES neighborhoods refer to census tracts in which >50 % of families lived below 185 % of the federal poverty level. Lower SES neighborhoods refer to census tracts in which ≤50 % of families lived below 185 % of the federal poverty level

At stores located in lower SES neighborhoods, the average number of eligible customers per hour (14) and the average number of interviews collected per hour (5.0) were slightly higher than at stores in higher SES neighborhoods (12 eligible customers per hour; 4.3 interviews per hour). Response rates did not differ by neighborhood SES.

Store visits beginning during weekday afternoon rush hour (4:00–6:00 pm) had the highest number of interviews collected per hour (5.5) and the highest response rate (40 %). Store visits beginning during weekday mornings and on weekends had the lowest number of interviews collected per hour (4.0 and 4.1, respectively). Weekends had the lowest response rates (32 %).

Daily temperature midpoints during data collection ranged from 0.5 to 23° Celsius, and approximately 20 % of data collection days had measurable precipitation (data not shown). There were no differences in response rates by daily temperature and precipitation.

### Characteristics of participants and non-participants

The gender distribution of participants was not significantly different from non-participants (*p* = 0.16), though the racial/ethnic distribution was significantly different (*p* < 0.01) (Table 3). Fifty-three percent of participants self-identified as White, compared to 61.3 % of non-participants that were presumed to be White. In contrast, 39.8 % of participants self-identified as Black, compared to 27.7 % of non-participants. Missing or unknown data for gender and race/ethnicity was more common among non-participants (i.e., when observed by data collectors) than when reported directly by participants; data collectors recorded 116 (or 5 %) non-participants’ gender as “don’t know” and 256 (or 10 %) non-participants’ race as “don’t know.” In addition, 70 participants (11 %) self-identified as more than one race/ethnicity, which was not an option for data collectors to select for non-participants.

**Table 3:** Characteristics of participants and non-participants

	Participants		Non-participants		
	N	%	N	%	*P*-value
Gender					
Male	343	56.2	1461	59.4	0.16
Female	267	43.8	999	40.6	
Race/Ethnicity					
White	288	53.0	1423	61.3*	<0.01
Black	216	39.8	643	27.7*	
Asian	18	3.3	158	6.8	
Hispanic	21	3.9	96	4.1	

* Statistically significantly different from participants at *p* < 0.01

Note: Tables includes both eligible and ineligible non-participants. Participants self-reported gender and race/ethnicity; data collectors assessed non-participants’ apparent gender and race/ethnicity. Participants reporting some other gender (*n* = 3) or with missing gender data (*n* = 3) and non-participants for whom data collectors did not know their gender (*n* = 116) excluded from analyses. Participants reporting some other race/more than one race (*n* = 70) or with missing race/ethnicity data (*n* = 3) and non-participants for whom data collectors recorded some other race (*n* = 2) or did not know their race (*n* = 254) excluded from analyses

## Discussion

After an iterative process involving weekly team meetings and field testing to improve and refine the recruitment protocol, study staff collected data from over 600 participants at 128 small and midsize food retailers during an 8-week period. During the pilot phase, study staff found it was important to quickly tell customers what was being asked of them in order to capture their attention. Once customers stopped, data collectors could fully explain the study and obtain consent to participate.

The response rate for this study (35 %) was similar to a recent study [12] of purchases outside corner stores across all areas of New York City (32.9 %), despite being conducted in a different geographic region of the country and including many non-traditional food/beverage retailers in the store sample. In contrast, two previous studies [3, 10] conducted outside corner stores in high-poverty neighborhoods and fast food restaurants reported higher response rates (range 53–63 %). Differences in study protocols, store type, and location may account for these differences.

A large number of individuals leaving the stores did not have a visible food, beverage, or store bag purchase, which likely resulted in a lower eligibility rate in the present study compared to studies of fast food purchases, for instance, where nearly all individuals exiting the store would be eligible. The present study did find response rates of 46–47 % outside of corner stores and dollar stores, which may be more comparable to these prior studies. The context of non-traditional food retailers, such as gas stations, may have contributed to lower response rates since customers perceive these store visits as brief errands on their way to their next destination. Neighborhood SES and weather did not appear to affect response rates, although data collectors were instructed to cancel shifts in the case of heavy rain or cold temperatures based on their own judgment.

While a higher response rate improves the generalizability of the sample to all customers, the number of interviews collected per hour also is helpful for staffing and resource considerations. The most productive data collection shifts were at food-gas marts, dollar stores, and shifts during the weekday afternoon rush hour. Data collection at other types of food stores not measured here (e.g., fast food restaurants, supermarkets) and other geographic locations (e.g., rural, suburban areas) may have greater productivity at different days or times. However, we believe the recruitment protocol described here could be adapted for use in these various settings. For example, in rural areas with greater distances between stores, researchers may wish to stay at a single location for a longer period of time to reduce time and resources spent traveling between locations.

### Strengths and limitations

To our knowledge, this is the first study of CI interviews in the food environment literature to collect and report data on non-participants to assess possible non-response bias and to examine possible environmental effects (e.g., neighborhood SES, weather) on response rates.

Use of the visual and verbal eligibility screens is both a strength and limitation of this study. Customers who placed food/beverage purchases in a purse, pocket, or backpack before exiting the store would be misclassified as ‘ineligible’ based on our protocol. On the other hand, an estimated 21 % of customers who passed the visual eligibility screen were ultimately determined to be ineligible for the study based on the verbal eligibility screen. Since refusals generally were not verbally screened for eligibility, some ineligible individuals were likely misclassified as refusals. Both types of misclassification would have impacted response rate calculations, albeit in different directions. Despite these limitations, the methodology and protocol developed here was a reasonable way to quickly and easily screen for eligibility.

Another limitation of our protocol is that participants self-reported their gender and race/ethnicity, whereas data collectors recorded their perception of non-participants’ gender and race/ethnicity. The comparisons between participants and non-participants should therefore be considered exploratory and interpreted with caution. The interview was conducted only in English; however, only 2.3 % of customers passing the visual eligibility screen were ineligible because of a language barrier. Weather data analyzed here included daily measures rather than weather at the time of data collection.

## Conclusions

To our knowledge, our analyses comparing CI participants to non-participants are the first published results of their kind in the food environment literature. The results indicate that there was no difference between the gender distribution of participants and non-participants, but that researchers should exercise caution when assuming that the sample that participates reflects the underlying race/ethnicity of the target population. It should be noted that although the team for baseline data collection was predominantly White and female, the team was able to recruit diverse participants to the study. Researchers should consider the ethnic composition of their study population and conduct interviews in multiple languages, if appropriate. This may require bilingual data collectors for each study language to be present on every shift.

Intercept interviews can be successfully used to recruit a diverse sample of customers at small and midsize food retailers. When developing protocols for community-based studies using CI interviews, consideration should be given both to response rates and efficient use of resources.

## Abbreviations

CI: Customer intercept
SES: Socioeconomic status
STORE: Staple foods ordinance evaluation
WIC: Special supplemental nutrition program for women, infants and children
